# Pioglitazone Improves Mitochondrial Function in the Remnant Kidney and Protects against Renal Fibrosis in 5/6 Nephrectomized Rats

**DOI:** 10.3389/fphar.2017.00545

**Published:** 2017-08-15

**Authors:** Li Sun, Quan Yuan, Tianhua Xu, Li Yao, Jiangmin Feng, Jianfei Ma, Lining Wang, Changlong Lu, Danan Wang

**Affiliations:** ^1^Department of Nephrology, The First Affiliated Hospital of China Medical University Shenyang, China; ^2^Department of Orthopedic Surgery, Shengjing Hospital of China Medical University Shenyang, China; ^3^Department of Immunology, China Medical University Shenyang, China

**Keywords:** chronic kidney diseases, peroxisome proliferator-activated receptor γ, pioglitazone, fibrosis, mitochondrial dysfunction

## Abstract

Pioglitazone is a type of peroxisome proliferator-activated receptor γ (PPARγ) agonist and has been demonstrated to be effective in chronic kidney diseases (CKD) treatment. However, the underlying mechanism involved in the renoprotection of pioglitazone has not been fully revealed. In the present study, the renoprotective mechanism of pioglitazone was investigated in 5/6 nephrectomized (Nx) rats and TGF-β1-exposed HK-2 cells. Pioglitazone attenuated renal injury and improved renal function, as examined by 24 h urinary protein, blood urea nitrogen and plasma creatinine in Nx rats. Renal fibrosis and enhanced expressions of profibrotic proteins TGF-β1, fibronectin and collagen I caused by Nx were significantly alleviated by pioglitazone. In addition, pioglitazone protected mitochondrial functions by stabilizing the mitochondrial membrane potential, inhibiting ROS generation, maintaining ATP production and the activities of complexes I and III, and preventing cytochrome C leakage from mitochondria. Pioglitazone also upregulated the expression levels of ATP synthase β, COX I and NDUFB8, which were downregulated in the kidney of Nx rats and TGF-β1-exposed HK-2 cells. Furthermore, pioglitazone increased fusion proteins Opa-1 and Mfn2 expressions and decreased fission protein Drp1 expression. The results imply that pioglitazone may exert the renoprotective effects through modulating mitochondrial electron transport chain and mitochondrial dynamics in CKD. Finally, these recoveries were completely or partly inhibited by GW9662, which suggests that these effects at least partly PPARγ dependent. This study provides evidence for the pharmacological mechanism of pioglitazone in the treatment of CKD.

## Introduction

Chronic renal failure (CRF), characterized by glomerulosclerosis and interstitial fibrosis, is the common end stage of all kinds of chronic kidney diseases (CKDs). CRF is usually associated with high mortality and the progression of CKD to CRF cannot be effectively intervened in clinic (Woo et al., 2015). Renal fibrosis, particularly tubulointerstitial fibrosis, is a typical feature in the progression of CKD which contributes to permanent loss of renal function (Iwano and Neilson, 2004; Inoue et al., 2015). Transforming growth factor-β1 (TGF-β1), an extensively studied profibrogenic cytokine, plays an important role in renal fibrosis in CKD (Wang et al., 2005; Lopez-Hernandez and Lopez-Novoa, 2012). It promotes fibrosis through several actions including increasing extracellular matrix (ECM) synthesis, decreasing ECM degradation, activating resident myofibroblast and inducing epithelial to mesenchymal transition (EMT) and inflammation (Lopez-Hernandez and Lopez-Novoa, 2012).

Mitochondria are essential eukaryotic cells organelles responsible for numerous important physiological processes such as energy generation, ROS production and cellular apoptosis (Newmeyer and Ferguson-Miller, 2003). Thus, mitochondrial dysfunction is detrimental to cells and organs because of the lack of energy supply and excessive ROS generation. As an organ with great demand of energy, kidney is abundant in mitochondria and mitochondrial dysfunction is one of the major factors in the pathogenesis of renal injury (de Cavanagh et al., 2007; Yuan et al., 2012). Clinical study also found that mitochondrial respiratory system was impaired in CKD patients (Granata et al., 2009). Treatments that improve mitochondrial functions have been proved to be beneficial for renal injury attenuation (Hui et al., 2017; Thomas et al., 2017; Zhao et al., 2017). Therefore, mitochondria may be a potent target for CKD treatment.

As a kind of effective antidiabetic drug, PPARγ agonists not only conquer diabetic nephropathy, but they are also considered potential therapeutic agents in non-diabetic CKD (Fogo, 2011). Previous studies have demonstrated that rosiglitazone could protect kidney from damage induced by several factors (Huang et al., 2013; Kumar et al., 2013; Korolczuk et al., 2014). Pioglitazone attenuated renal ischemia-reperfusion injury through its anti-inflammatory and antioxidant effects (Reel et al., 2013; Zou et al., 2013), reduced kidney damage in diabetic rats through increasing glomerular podocalyxin protein expression (Peng et al., 2014) and provided renoprotection by interfering with the renin-angiotensin system and profibrotic proteins (Ochodnicky et al., 2014). In addition, mitochondria are a target of PPARγ agonists. They can improve mitochondrial functions including mitochondrial respiration, ROS production and ATP generation in various diseases (Patel et al., 2017). Furthermore, PPARγ agonists can also modulate mitochondrial metabolism proteins such as dynamin related protein 1 (Drp1) and mitochondrial fission protein 1 (Fis1) (Zolezzi et al., 2013; Chuang et al., 2016). Particularly, rosiglitazone PPARγ-dependently protects podocyte from aldosterone-induced injury and restores mitochondrial function (Zhu et al., 2011) and pioglitazone protected against aging-related renal injury by improving mitochondrial functions (Yang et al., 2009).

Our previous study showed that pioglitazone benefited renal failure through activation of the antioxidative system and inhibition of angiogenesis in the remnant kidney (Sun et al., 2016). In the present study, we aim to continue to investigate the pharmacological mechanisms of pioglitazone in the 5/6 nephrectomized (Nx) rat model from the angle of fibrosis and mitochondrial functions.

## Materials and Methods

### Primary Antibodies

Cytochrome c (Cyt C) antibody was purchased from Boster (Wuhan, China). TGF-β antibody, collagen I antibody, PPARγ antibody, ATP synthase β (ATP β) antibody, Cyt C oxidase subunit I (COX I) antibody, NDUFB8 antibody, optic atrophy 1 (Opa1) antibody, mitofusin (Mfn)2 antibody, dynamin-related protein 1 (Drp1) antibody, voltage-dependent anion-selective channel (VDAC) antibody and β-actin antibody were purchased from Bioss (Beijing, China). Fibronectin antibody was purchased from Sangon Biotech (Shanghai) Co., Ltd. (Shanghai, China).

### Animals

Male Sprague-Dawley (SD) rats (200–220 g) purchased from the Experimental Animal Center of China Medical University (Shenyang, China) were housed in a 12/12 h light-dark cycle, with *ad libitum* standard food experimental rodents and water. The rats were randomly divided into four groups (*n* = 6 for each group): (1) sham; (2) 5/6 nephrectomy (Nx); (3) Nx+ pioglitazone (Pio); and (4) Nx+ Pioglitazone +GW9662 (GW). The experimental model of CKD was established according to the previous description (Tapia et al., 2012). Briefly, under anesthesia with 30 mg/kg sodium pentobarbital, 5/6 Nx was performed by removal of the right kidney and lower and upper thirds of the left kidney. Seven days after the surgery, rats in the Pio and GW groups received pioglitazone (10 mg/kg) daily by gavage for 8 weeks. Rats in the GW group received GW9662 (1 mg/kg) 1 h before administration of pioglitazone daily by intraperitoneal injection. All the animal experiments were performed in accordance with the Animal Care Guidelines for the Care and Use of Laboratory Animals and the protocol was approved by the Institutional Animal Ethics Committee of China Medical University.

### Assessments of Urine and Blood

Twenty-four-hour urine and serum samples were collected 8 weeks after treatment. Twenty-four-hour proteinuria, blood urea nitrogen (BUN) and serum creatinine levels were determined using commercial proteinuria assay kits purchased from Nanjing Jiancheng Bioengineering Institute (Nanjing, China).

### Histological Examination

Renal tissues from rats were fixed with 4% paraformaldehyde, embedded in paraffin, and cut into 5-μm-thick section. After dewaxing and gradient ethanol hydration, kidney sections were stained with periodic acid schiff (PAS) reagent (Baso Diagnostic Inc., Zhuhai, China) or Masson’s trichrome solution. The sections were then observed under an optic microscopy (DP73; Olympus, Tokyo, Japan).

### Cell Culture and Treatment

The primary human proximal tubular cell line HK-2 was obtained from the Cell Bank of Type Culture Collection of Chinese Academy of Sciences (Shanghai, China). HK-2 cells were cultured in Dulbecco’s Modified Eagle’s Medium (DMEM) (Gibco, Grand Island, NY, United States) supplemented with 10% fetal calf serum (FBS; Gibco) at 37°C in a humidified 5% CO_2_ incubator. HK-2 cells were incubated with pioglitazone 5 μM with or without TGF-β1 (2 ng/mL) for 24 h. In addition, 1 μM GW5662 was added alone with pioglitazone to evaluate whether the effect of pioglitazone on the TGF-β1-exposed cells was PPARγ dependent.

### Mitochondria Isolation and Mitochondrial Function Determination

Mitochondria were isolated from the fresh kidney using a mitochondrial isolation kit (Beyotime Institute of Biotechnology, Haimen, China) according to the manufacture’s instruction. The protein concentration of the pellet mitochondria was measured using a Bicinchoninic Acid (BCA) protein assay kit (Beyotime).

Mitochondrial function was evaluated by determining mitochondrial membrane potential (MMP), intracellular reactive oxygen species (ROS) generation, ATP production and the activities of mitochondrial complexes I and III. These parameters were determined by the commercial kits for MMP assay (Beyotime Institute of Biotechnology, Haimen, China) using JC-1 method, ROS assay (Nanjing Jiancheng Bioengineering Institute, Nanjing, China) using DCF-DA method, ATP determination (Nanjing Jiancheng) and complexes I and III activities assay (Genmed, Shanghai, China) following the instructions.

### Western Blotting

Kidney tissues and HK-2 cells were homogenized in cooled radioimmunoprecipitation buffer (RIPA, Beyotime) supplemented with 1% PMSF (Beyotime) and centrifuged at 12,000 *g* for 10 min at 4°C. Protein samples in each group were loaded on SDS-polyacrylamide gel and performed electrophoresis. Target proteins were transferred onto polyvinylidene fluoride membranes (Millipore, Billerica, MA, United States) and blocked in 5% non-fat milk at room temperature for 1 h. The membranes were incubated with primary antibodies at 4°C overnight and then incubated with HRP conjugated secondary goat anti rabbit antibody (1: 5000; Beyotime) at 37°C for 45 min. After washing, immunoblots were developed using the enhanced chemiluminescence reagent (Beyotime) and exposed on Fuji Rx 100 X-ray film (Fuji Photo Film, Tokyo, Japan). β-actin was used as the internal control and gray values of the blots was analyzed with Gel-Pro-Analyzer software (Media Cybernetics, Bethesda, MD, United States).

### Statistical Analysis

Data are presented as mean ± standard deviation (SD). They were analyzed by one-way analysis of variance with a subsequent LSD test using the software SPSS 19.0 (IBM, New York, NY, United States). A *P*-value less than 0.05 was considered significant.

## Results

### Pioglitazone Restored Renal Structure and Function in Nx Rats

**FIGURE 1:**
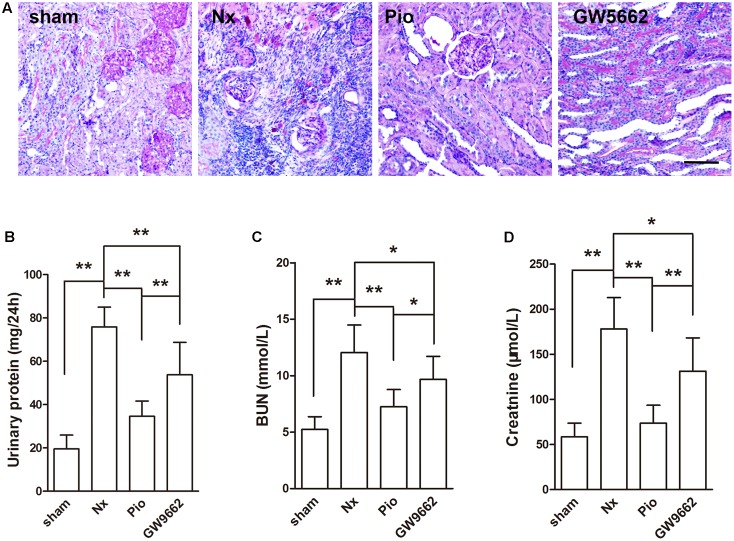
Effects of pioglitazone on renal structure and renal function in Nx rats. **(A)** PAS staining showed tubular atrophy and tubular dilation in the kidney of Nx rats, and pioglitazone treatment attenuated these injuries. Scale bar: 100 μm **(B)** Urinary protein excretion rate, **(C)** blood urea nitrogen (BUN) and **(D)** plasma creatinine were increased in Nx rats and pioglitazone treatment restored the increase. The therapeutic effect of pioglitazone was inhibited by the PPARγ inhibitor GW9662. *n* = 6. ^∗^*P* < 0.05, ^∗∗^*P* < 0.01, compared with the Nx group.

The renal injury was examined using PAS staining. **Figure 1A** showed that no obvious damage in the kidney of sham group. In the kidney of Nx rats, serious glomerular sclerosis and tubulointerstitial fibrosis were found. These injuries were attenuated after pioglitazone treatment, PPAR γ inhibitor GW9662 blocked the effects of pioglitazone. Proteinuria and levels of BUN and Cr were examined to reflect the renal functions. As shown in **Figures 1B–D**, 5/6 nephrectomy significantly increased 24 h proteinuria, BUN and Cr in rats (*P* < 0.05 compared with the sham group), which indicated the damaged renal functions in Nx rats. Consistent with the results of PAS staining, pioglitazone improved renal functions as evidenced by the markedly reduced 24 h proteinuria, BUN and Cr compared with that in the Nx rats (*P* < 0.01). Although not completely, GW9662 inhibited the effects of pioglitazone.

### Pioglitazone Attenuated Renal Fibrosis in Nx Rats

**FIGURE 2:**
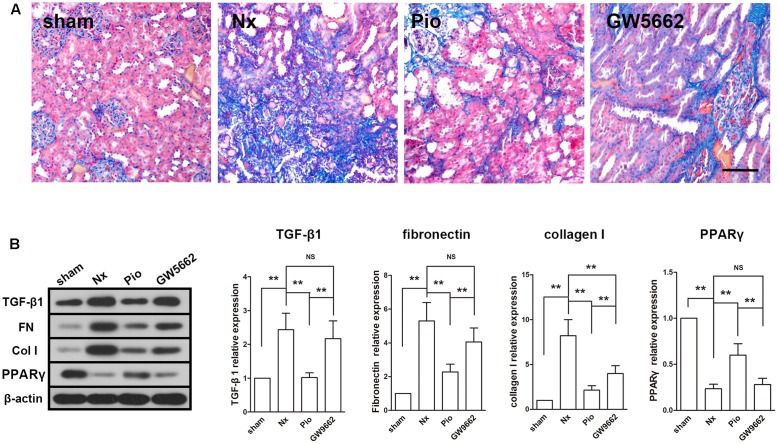
Effects of pioglitazone on renal fibrosis and profibrotic proteins in the kidney of Nx rats. **(A)** Masson’s staining showed large fibrotic area in the kidney of Nx rats and pioglitazone significantly reduced the fibrotic area. Scale bar: 100 μm. **(B)** The increased expressions of profibrotic proteins TGF-β1, fibronectin and collagen I in the kidney of Nx rats were markedly decreased after pioglitazone treatment. These effects were at least partly PPARγ dependent. *n* = 6. ^∗^*P* < 0.05, ^∗∗^*P* < 0.01.

Masson staining showed a large area of fibrosis in the kidney of Nx rats and the fibrotic area was significantly reduced in pioglitazone treated rats (**Figure 2A**). However, the fibrosis in GW9662 group was as large as that in the Nx group, which indicated that GW9662 inhibited the anti-fibrotic effect of pioglitazone. Correspondingly, the expression levels of fibrosis-associated proteins were dramatically upregulated in the kidney of Nx rats compared with that in the sham rats (*P* < 0.01, **Figure 2B**). Treatment with pioglitazone significantly inhibited these upregulations and this effect could also be inhibited by GW9662. These effects were consistent with the expression of PPARγ, which suggested that the anti-fibrotic effects may be PPARγ-dependent.

### Pioglitazone Improved Mitochondrial Functions in the Kidney of Nx Rats

**FIGURE 3:**
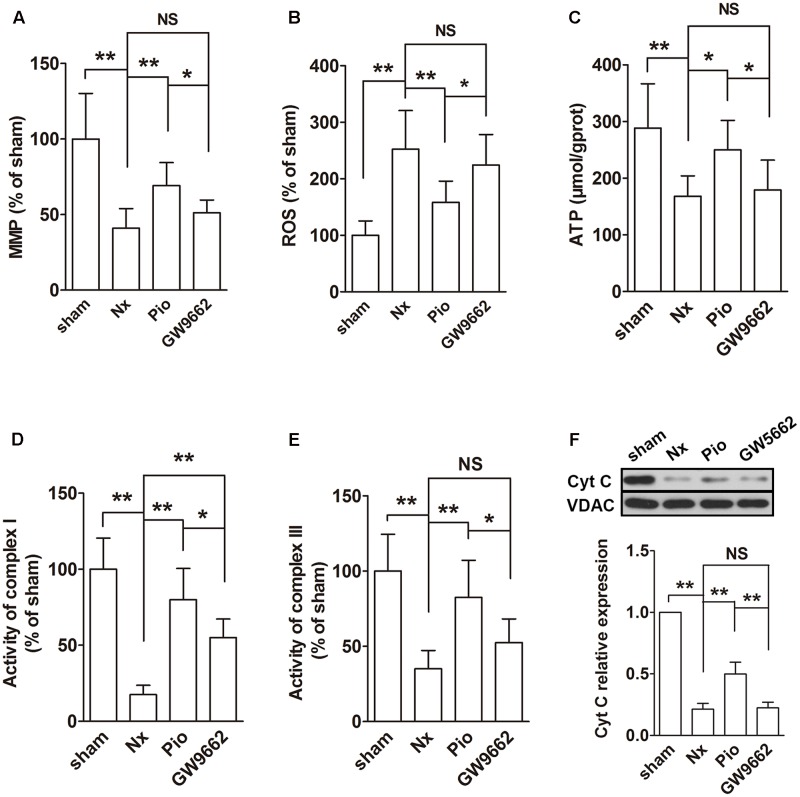
Effects of pioglitazone on mitochondrial function in the kidney of Nx rats. Obvious mitochondrial dysfunction was found in the kidney of Nx rats as evidenced by decreased MMP **(A)**, increased ROS generation **(B)** and reduced ATP production **(C)**, complexes I and III activities **(D,E)** and mitochondrial Cyt C expression **(F)**. Pioglitazone improved mitochondrial function by restoring these parameters. *n* = 6. ^∗^*P* < 0.05, ^∗∗^*P* < 0.01, ^NS^*P* > 0.05.

The functions of mitochondria were evaluated using MMP, ROS and ATP production, activities of complexes I and III and mitochondrial Cyt C expression. In the remnant kidney, we found a significant decline in MMP, a dramatic increase in ROS production and a reduced ATP production compared with that in the sham group (**Figures 3A–C**, *P* < 0.01). In addition, the activities of complexes I and III was inhibited and the Cyt C expression in mitochondria was downregulated in the remnant kidney of rats (**Figures 3D–F**). Treatment with pioglitazone restored these changes, indicating that pioglitazone could improve mitochondrial functions in the kidney of Nx rats.

### Pioglitazone Modulated Mitochondrial Electron Transport Chain Mitochondrial Metabolism in the Kidney of Nx Rats

**FIGURE 4:**
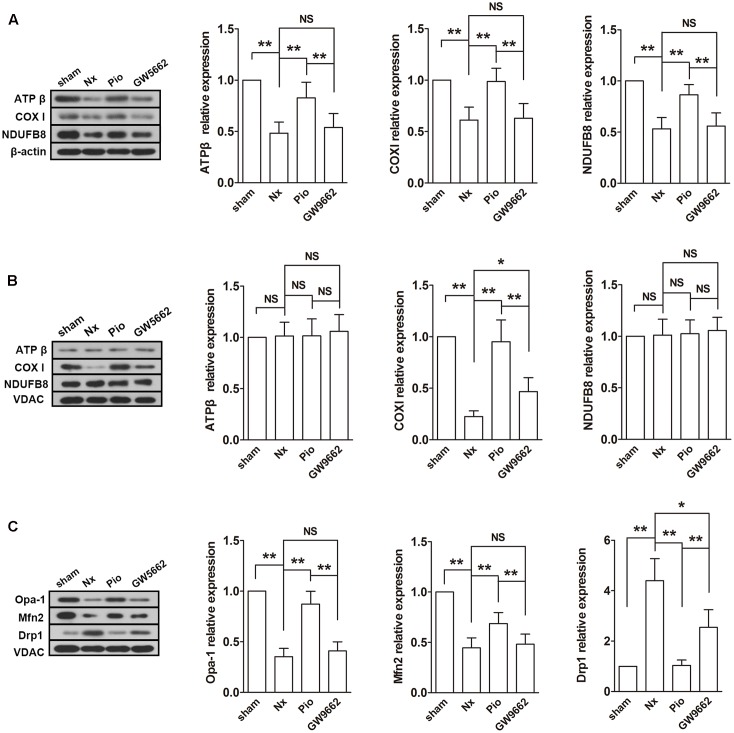
Effects of pioglitazone on ETC and mitochondrial dynamics in the kidney of Nx rats. The ETC proteins COX I was found to be decreased after surgery in both of isolated mitochondria **(A)** and tissue **(B)**. The expression of ATP β and NDUFB8 were only found to be reduced in the renal tissue. Mitochondrial fusion proteins Opa-1 and Mfn2 were decreased and fusion protein Drp1 was increased in isolated mitochondria from the kidney of Nx rats **(C)**. Pioglitazone treatment restored these changes. *n* = 6. ^∗^*P* < 0.05, ^∗∗^*P* < 0.01, ^NS^*P* > 0.05.

Subsequently, the expressions of mitochondrial electron transport chain (ETC) protein in the renal tissues and isolated mitochondria were examined. As shown in **Figure 4A**, the nuclear-encoded proteins ATPβ and NDUFB8, and the mitochondrial-encoded protein COX I were decreased in the renal tissue. However, the expression levels of ATPβ and NDUFB8 were not changed in the isolated mitochondria (**Figure 4B**). As expected, pioglitazone upregulated these changed proteins, which indicated that pioglitazone may improve mitochondrial functions through regulating ETC proteins.

Fusion and fission are two important processes in mitochondrial metabolism. In mammalian cells, Opa-1 and Mfn are the main regulators of mitochondrial fusion and Drp-1 is the main fission protein. To further elucidate the mitochondrial protective mechanisms of pioglitazone, the expression levels of these proteins were assessed. As illustrated in **Figure 4C**, the expression levels of Opa-1 and Mfn2 were downregulated and the expression level of Drp-1 was upregulated in the remnant kidney of Nx rats (*P* < 0.01 compared with the sham group), which indicated the rate of fusion was decreased and the rate of fission was increased. Treatment with pioglitazone significantly upregulated Opa-1 and Mfn2 and downregulated Drp-1. The regulation of mitochondrial metabolism may also contribute to its mitochondrial protection.

Most of the effect of pioglitazone was inhibited by GW9662 although the inhibitory effects were not complete in some parameters such as the activity of complex I and the expression of Drp1.

### Effect of Pioglitazone on Fibrosis-Associated Proteins in TGF-β1-Exposed HK2 Cells

**FIGURE 5:**
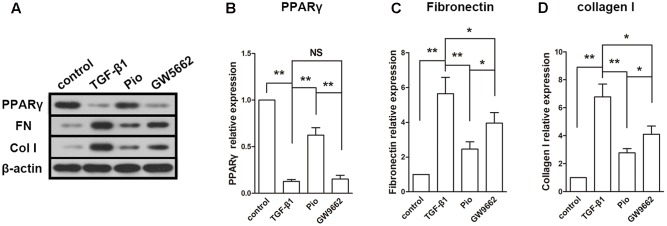
Effects of pioglitazone on profibrotic proteins in TGF-β1-exposed HK-2 cells. **(A)** Typical protein blots of PPARγ and profibrotic proteins. **(B)** Protein expression of PPARγ, **(C)** protein expression of fibronectin, **(D)** protein expression of collagen I. Pioglitazone inhibited profibrotic proteins expression induced by TGF-β1 in HK-2 cells. *n* = 3. ^∗^*P* < 0.05, ^∗∗^*P* < 0.01, ^NS^*P* > 0.05.

The expression of PPARγ was inhibited by TGF-β1 and upregulated by pioglitazone. GW9662 suppressed the effect of pioglitazone. In the detection of fibrosis-associated proteins, we found that TGF-β1 induced dramatic increases in the expressions of fibronectin and collagen I. In agreement with the results in the *in vivo* study, pioglitazone treatment markedly inhibited the upregulation of fibronectin and collagen I, and this effect was partly blocked by GW9662 (**Figures 5A–D**). These effects on fibrotic proteins paralleled to that on the expression of PPARγ.

### Pioglitazone Improved Mitochondrial Functions and Regulated Mitochondrial Metabolism in TGF-β1-Induced HK-2 Cells

**FIGURE 6:**
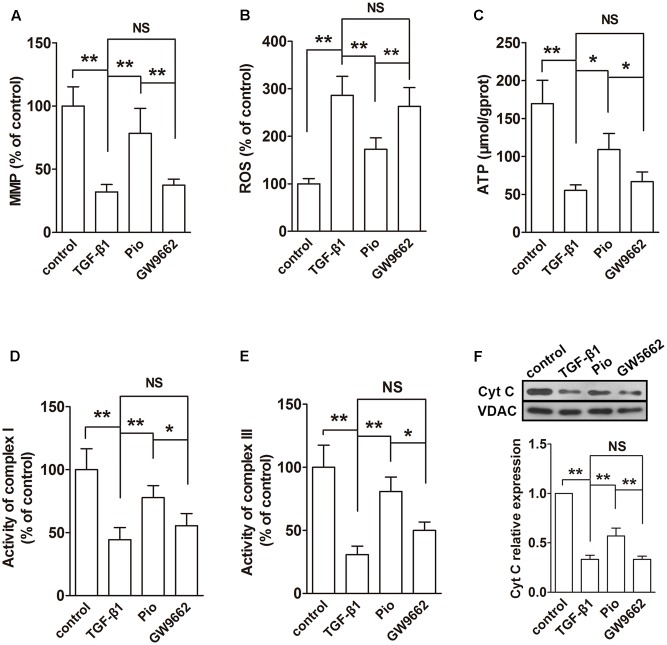
Effects of pioglitazone on mitochondrial function in TGF-β1-exposed HK-2 cells. TGF-β1 induced mitochondrial dysfunction in HK-2 cells as evidenced by decreased MMP **(A)**, increased ROS generation **(B)** and reduced ATP production **(C)**, complexes I and III activities **(D,E)** and mitochondrial Cyt C expression **(F)**. Pioglitazone improved mitochondrial function by restoring these parameters. *n* = 3. ^∗^*P* < 0.05, ^∗∗^*P* < 0.01, ^NS^*P* > 0.05.

**FIGURE 7:**
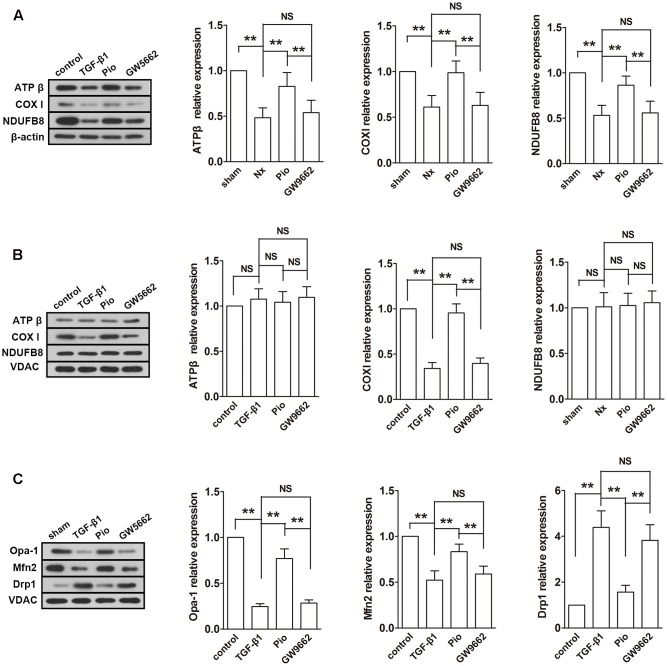
Effects of pioglitazone on ETC and mitochondrial dynamics in TGF-β1-exposed HK-2 cells. TGF-β1 induced downregulation of ETC proteins ATP β, COX I and NDUFB8 in the renal tissue **(A)** and downregulation of COX I in isolated mitochondria **(B)**. In addition, mitochondrial fusion proteins Opa-1 and Mfn2 were decreased and fusion protein Drp1 was increased in isolated mitochondria from the TGF-β1-exposed HK-2 cells **(C)**. Treatment of pioglitazone normalized the expressions of these proteins in a PPARγ-dependent manner. *n* = 3. ^∗∗^*P* < 0.01, ^NS^*P* > 0.05.

The effects of pioglitazone on mitochondrial was also examined *in vitro* in TGF-β1-induced HK-2 cells. Similar to the observation in the kidney of Nx rats, TGF-β1 induced reduced MMP, ATP production, activities of complexes I and III and expression of mitochondrial Cyt C and increased ROS production (**Figures 6A–F**). In the examination of the expressions of ETC proteins, TGF-β1 significantly lowered the expression levels of ATP synthase β, COXI and NDUFB8 in the whole cells (**Figure 7A**) and the expression level of mitochondrial-encoded COX I in the isolated mitochondria (**Figure 7B**). In addition, mitochondrial fusion protein Opa-1 and Mfn2 were downregulated and fission protein Drp1 was upregulated in mitochondria induced by TGF-β1, which was similar to that in the *in vivo* study (**Figure 7C**). As expected, addition of pioglitazone inhibited these effects of TGF-β1, and GW9662 could block the effects of pioglitazone.

## Discussion

The present study demonstrated that PPARγ agonist pioglitazone attenuated renal injury and tubulointerstitial fibrosis evidenced by the improved renal functions, reduced fibrotic areas and downregulated profibrotic proteins. In addition, pioglitazone alleviated mitochondrial dysfunction and modulated mitochondrial ETC and dynamic proteins in the remnant kidney.

Extracellular matrix deposition is a common feature of tissue fibrosis. As an early biomarker of fibrosis, TGF-β1 induces synthesis of ECM components such as collagen and fibronectin (Leask and Abraham, 2004). Previous study has demonstrated that pioglitazone inhibited TGF-β1-induced fibronectin mRNA expression in mouse mesangial cells (Guo et al., 2004). In agreement with this finding, we found that pioglitazone not only decreased the expression of TGF-β1 in the remnant kidney, but also downregulated the TGF-β1-induced collagen I and fibronectin expressions in HK2 cells, which indicated that pioglitazone may both target to TGF-β1 and its downstream profibrotic proteins. In addition, we found that PPARγ antagonist GW9662 completely inhibited pioglitazone-induced expression of PPARγ, but has not totally suppressed the effects of pioglitazone on all the profibrotic proteins we detected. This finding suggests that the anti-tubulointerstitial fibrosis effects of pioglitazone may be through both PPARγ-dependent and -independent pathways. The detailed mechanisms need to be investigated in further studies.

Mitochondria are important cellular organelles that are responsible for numerous physiological processes such as energy production, oxidative phosphorylation and Ca^2+^ homeostasis. In this study, 5/6 nephrectomy induced renal injury in rats, accompanied by mitochondrial dysfunction, as evidenced by the increased ROS generation, decreased MMP and ATP production, reduced activities of complexes I and III, and downregulated Cyt C expression in mitochondria in the remnant kidney. These observations suggest the damage to the mitochondrial membrane, respiratory chain complex inhibition and leakage of Cyt C from the mitochondria. Pioglitazone restored these changes. *In vitro* studies provide compelling evidence that TGF-β1 induced mitochondrial dysfunction in HK-2 cells. Energy supply is the major mission of mitochondria. In mitochondria, ATP is produced by ETC using the proton gradient via oxidative phosphorylation (Bertram et al., 2006). In this study, three typical ETC proteins, ATPβ, complex I subunit NDUFB8 and COX I were examined. NDUFB8 and COX I are the enzymes in mitochondrial ETC. ATPβ, also called complex V, is important in ATP production through oxidative phosphorylation. We found that the expression levels of all the three proteins in the whole cell were downregulated both in the remnant kidney and TGF-β1-induced HK-2 cells. However, in the isolated mitochondria, two nuclear DNA-encoded protein NDUFB8 and ATPβ were unchanged. Unfortunately, we cannot make exact conclusion from our data, this discrepancy may due to the auto-regulation of the transport mechanisms or may be attributed to the time point we detected as discussed in a previous study (Hui et al., 2017). Anyhow, pioglitazone protected mitochondrial function from ETC damage, thereby restoring ATP production, and this effect is, at least partly, PPARγ-dependent. These results indicate that the mitochondria protective effects may contribute to the renoprotection of pioglitazone.

Maintaining homeostasis of cells and mitochondrial functions require a dynamic balance between mitochondrial fission and fusion. The imbalance of the mitochondrial dynamics largely contributes to the pathogenesis of the tissues (Archer, 2013). Mitochondrial fission and fusion are regulated by several dynamin-related GTPase proteins. Drp1, a GTPase localized in the cytosol, is the key mediator in mitochondrial fission in a variety types of cells (Smirnova et al., 2001). During fission, Drp1 is recruit by its receptors to outer membrane and oligomerized to promote membrane constriction and scission (Smirnova et al., 2001). For mitochondrial fusion, the inner membrane fusion and the outer membrane fusion are regulated by different proteins. Mfn1 and 2 are responsible for outer membrane fusion and Opa1 is responsible for inner membrane fusion (Santel and Fuller, 2001). In this study, the mitochondrial dynamics were found to be pathologically remodeled in the remnant kidney and TGF-β1-induced HK-2 cells, as evidenced by downregulated expression of mitochondrial fusion proteins Mfn2 and Opa1 and increased expression of fission protein Drp1. This finding is in agreement with Hui et al. (2017) study. However, Aparicio-Trejo OE et al reported an opposite finding in the same model (Aparicio-Trejo et al., 2017). This discrepancy may be attributed to the different time point to detection–we and Hui et al. (2017) performed the detection 8 and 4 weeks after the model establishment, respectively, whereas Aparicio-Trejo et al. (2017) did the examination 24 h after the nephrectomy. In their study, the shift of mitochondrial dynamics to fusion was as a response to energy demand, which may be a compensatory mechanism to complement mitochondrial dysfunction. In our studies, after long time damage, the mitochondria lost its auto-compensation as evidenced by the increased expression of fission protein. Excessive fission is a key factor of mitochondrial dysfunction and inhibitor of fission has been developing as a therapeutic strategy for mitochondrial diseases (Reddy, 2014). Here we found that pioglitazone PPARγ-dependently normalized the expression levels of mitochondrial dynamic proteins, which indicates that pioglitazone exerts its mitochondrial protective effects through modulating mitochondrial dynamics.

Although the present study did not reveal exact molecular target of pioglitazone, we can deduce that the modulation of TGF-β1expression and mitochondrial dysfunction may be reciprocal. The interaction between TGF-β1 signaling and mitochondria has been demonstrated in the previous studies (Willaert et al., 2012; Jain et al., 2013). Corcoran et al demonstrated that mitochondria were involved in the amplification of TGF-β1 signaling in renal epithelial cells (Corcoran et al., 2013), suggesting mitochondria may contribute to the TGF-β1-induced renal fibrosis. In our findings, TGF-β1 expression and mitochondrial functions were both recovered after pioglitazone treatment, which indicates that pioglitazone may protects kidney from fibrosis through improving mitochondrial functions.

In summary, pioglitazone attenuates mitochondrial dysfunction in the kidney of CKD rat model. This effect may contribute to its effects on renal function improvement and renal fibrosis inhibition. Our study provides new evidence of the anti-fibrotic mechanisms of pioglitazone and suggests that pioglitazone may be effective in preventing renal fibrosis in CKD treatment.

## Author Contributions

The study was designed by LS and DW. The article was written by LS. The experiments was performed by LS, QY, TX, LY, JF, JM, LW, and CL. The data was analyzed by LS, QY, and TX.

## Conflict of Interest Statement

The authors declare that the research was conducted in the absence of any commercial or financial relationships that could be construed as a potential conflict of interest.
